# Pulmonary Sequestration with Renal Aplasia and Elevated SUV Level in PET/CT

**DOI:** 10.1155/2012/276012

**Published:** 2012-12-12

**Authors:** Serdar Şen, Nilgün Kanlıoğlu Kuman, Ekrem Şentürk, Engin Pabuşcu, Ertan Yaman

**Affiliations:** ^1^Thoracic Surgery Department, Faculty of Medicine, Adnan Menderes University, Aydın 09000, Turkey; ^2^Thoracic Surgery Department, Osmaniye State Hospital, Toprakkale, Turkey; ^3^Thoracic Surgery Department, Çorum State Hospital Corum, Turkey

## Abstract

Extralobar sequestration with other bronchopulmonary malformations is commonly seen; however, the association of extralobar sequestration with renal aplasia is very rare. A 75-year-old female patient was admitted with back pain. Ultrasonography revealed aplasia of the left kidney and tomography showed 6 × 4.5 cm sized tumor in the left hemithorax at the posterobasal area. The lesion has focally increased glycolytic activity (SUVmax: 3.2) at the left upper pole on positron emission tomography scan (PET/CT). Sequestrectomy was performed after the confirmation by frozen section that the lesion was benign and of extrapulmonary sequestration. No complication occurred during postoperative and 50-month follow-up period.

## 1. Introduction

Pulmonary sequestration (PS) is a rare anomaly in the spectrum of congenital bronchopulmonary malformations that occur by any given impairment of embryonic development. 

Two forms of pulmonary sequestration are described depending on whether or not the abnormal lung tissue possesses its own pleural covering, such as intralobar and extralobar sequestration. The ratio of intralobar to extralobar sequestration is about 3 : 1 [1].

Extralobar pulmonary sequestration (ELS) has its own sac that is anatomically separated from the rest of the lung and usually obtains its blood supply from systemic vessels [2]. The arterial supply to 80% ELS comes directly from the thoracic or abdominal aorta, with approximately 15% receiving blood via another systemic artery and 5% via the pulmonary artery [3].

## 2. Case Report

**Figure 1:**
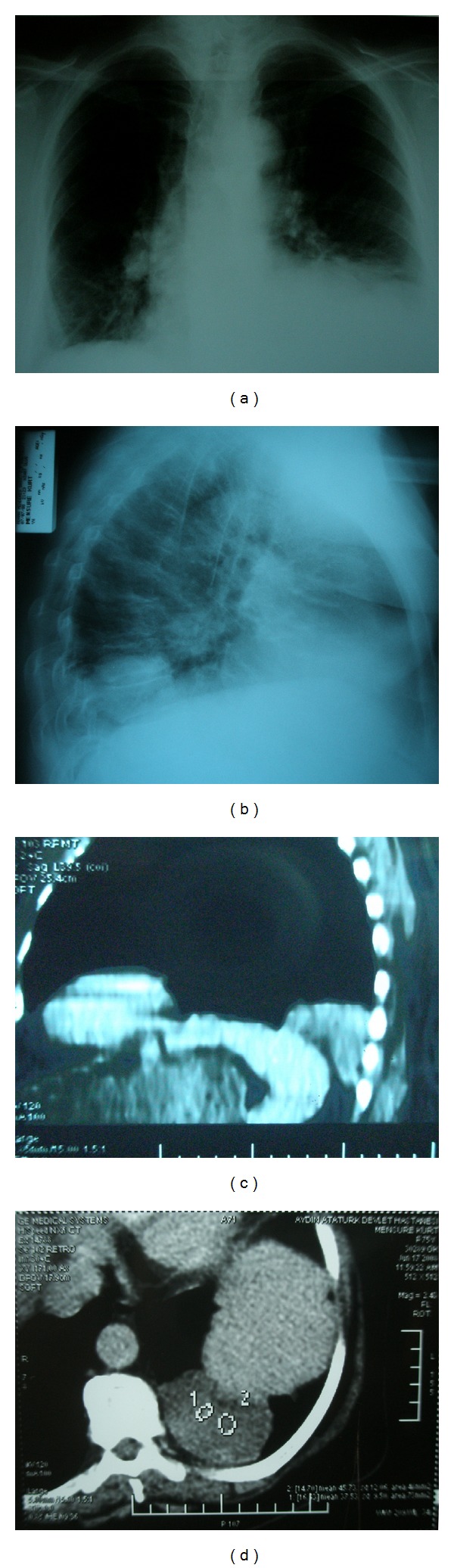
(a and b) Lesion can be seen in left hemithorax at inferior zone above diaphragm in chest radiographies (c and d) Thorax CT showing thatthe lesion has microcalcifications and close relationship with diaphragm and posterior costophrenic sinus.

 A seventy-five-year-old female patient was admitted to our hospital with back and abdominal pain. Routine laboratory tests were in normal limits and yielded no differential diagnosis. There was a tenderness in right upper abdomen in physical examination. Abdominal ultrasonography revealed aplasia of left kidney and an increased density was observed in the left lower zone on chest radiography. The patient had not suffered from kidney related disease formerly. Chest tomography (CT) showed 6 × 4.5 cm sized tumor with regular shape that had millimetric calcification in the left hemithorax in the lower lobe in posterobasal area (Figure 1).

**Figure 2:**
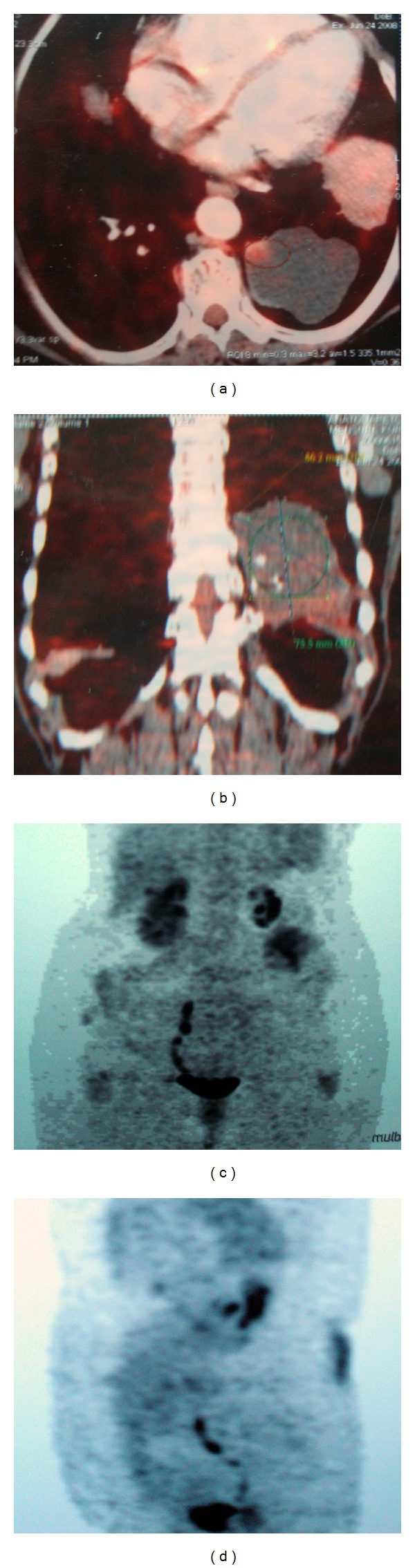
(a and b) Focally increased glycolytic activity in the lesion detected on PET CT. (c and d) Decreased glycolytic activity of the left kidney was seen on PET CT.

 Homogeneous and hypodense tumor has focal increase of glycolytic activity (SUVmax; 3.2) at the left upper pole of the lesion on PET/CT (Figure 2). 

**Figure 3:**
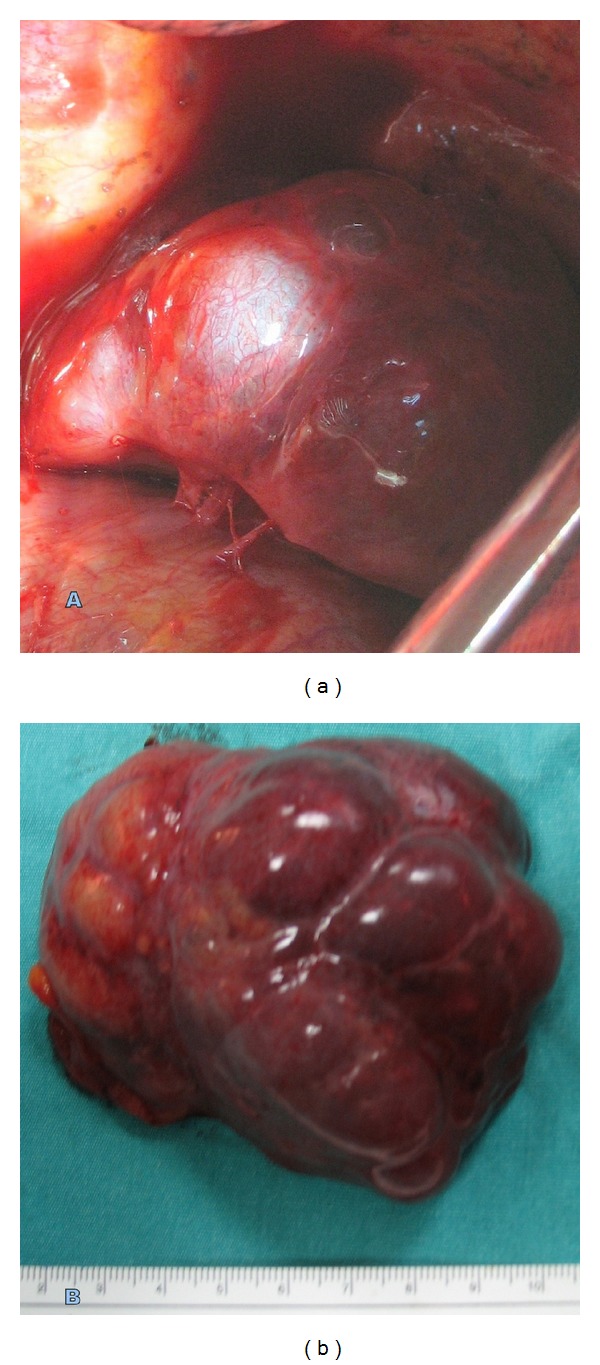
(a) Blood supply of the lesion comes from the left diaphragm. (b) Smooth, lobulated, sequestrated lung tissue is seen on macroscopic image.

A cystic, 8 cm sized intrathoracic extrapulmonary lesion with benign characteristics was observed in operation. Sequestrectomy was performed following confirmation that the lesion was benign and was of extrapulmonary sequestration with frozen section examination. Arterial supply was from the centrum tendineum of the left diaphragm. (Figure 3). 

**Figure 4:**
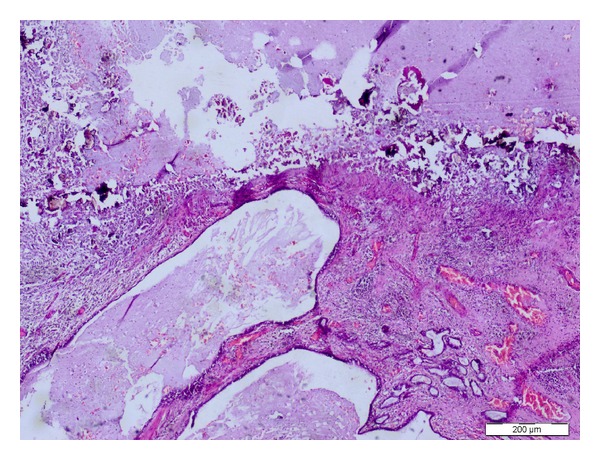
Microscopic examination revealed ectatic bronchial structures, inflammation, and microcalcification (HE, ×100).

 Cystic sequestrectomy material was filled with mucous and haemorrhagic fluid. Microscopic examination revealed ectatic bronchial structures in which there were overall inflammation and microcalcification (Figure 4). 

There was no evidence of malignant transformation. No complication occurred during early postoperative and 50-month follow-up period.

## 3. Discussion

More than 60% of patients with ELS have coexisting congenital anomalies and congenital diaphragmatic hernia that consists of the most common anomaly of these (16%). About 25% of ELS were found in association with other congenital lung abnormality such as hypoplasia, congenital cystic adenomatoid malformation (CCAM), congenital lobar emphysema, or bronchogenic cyst [3]. In the present study we found unilateral renal aplasia, which is an extremely rare experience. Aplasia of left kidney revealed via abdominal ultrasonography and decreased glycolytic activity viewed on PET CT. As to our knowledge, there is no kidney aplasia associated with ELS in related publications. 

Clinical manifestations of ELS are quite variable. Recurrent infections and respiratory distress or an asymptomatic mass can be clinically manifested [4]. Also back pain can be observed if torsion of ELS was occurred [5]. In our case there was no evidence of torsion; however, back pain might depend on diaphragmatic irritation or preexisting abdominal illness. 

In some adults, ELS may occur in an unusual mediastinal location, which might be suspected to be malignancy [1]. PET/CT examination showed moderate SUV elevation in a part of the lesion which depended on chronic inflammation in our case and which was initially considered as a malignant degeneration. 

ELS was diagnosed preoperatively in 9% of the cases [1]. Pulmonary angiography, magnetic resonance imaging, computed tomography scanning, bronchography, and ultrasonography have all been used in selected cases to confirm preoperative diagnosis [1]. Scar tissue due to recurrent infections may obscure the artery in sequestration. These adhesions can be very dense, and scar tissue may mimic the artery [6]. 

Typical radiologic appearance is a homogeneous soft-tissue mass in the lower hemithorax [7]. The other localizations of the ELS were mediastinum and interior of diaphragm, although localizations below the diaphragm are seldom [7]. Numerous reports have described severe complications due to pulmonary sequestration, such as fungal infection, tuberculosis, fatal hemoptysis, massive hemothorax, cardiovascular problems, and even malignant degeneration of ELS [8]. Main treatment of pulmonary sequestration is resection, especially in symptomatic cases [1]. The resection can be made due to thoracotomy or video-assisted thoracic surgery (VATS) [5]. The intraoperative blood loss is relatively high in VATS series because of the dense and wide adhesions and inflammation especially in the cases with pulmonary abscess. Serious hemorrhage and even death have been reported when this condition is not recognized at surgery [6].

Some groups have also reported that the use of coil embolisation in infants is a less invasive manner to eliminate the feeding artery [6]. Overlook of a large systemic blood vessel may result in lethal hemorrhage due to retraction of the vessel below the diaphragm [5].

We prefer the resection of pulmonary malformations in order to establish a definitive diagnosis and also to prevent potentially serious complications and late infections.
